# Evaluation of surfactin synthesis in a genome reduced *Bacillus subtilis* strain

**DOI:** 10.1186/s13568-019-0806-5

**Published:** 2019-06-12

**Authors:** Mareen Geissler, Ines Kühle, Kambiz Morabbi Heravi, Josef Altenbuchner, Marius Henkel, Rudolf Hausmann

**Affiliations:** 1 Department of Bioprocess Engineering (150k), Institute of Food Science and Biotechnology (150), University of Hohenheim, Fruwirthstr. 12, 70599 Stuttgart, Germany; 2 Institute of Industrial Genetics, University of Stuttgart, Allmandring 31, 70569 Stuttgart, Germany

**Keywords:** Lipopeptide, Biosurfactant, Strain development, Genome reduction, Anaerobic

## Abstract

Strain engineering is often a method of choice towards increasing the yields of the biosurfactant surfactin which is naturally synthesized by many *Bacillus* spp., most notably *Bacillus subtilis*. In the current study, a genome reduced *B. subtilis* 168 strain lacking 10% of the genome was established and tested for its suitability to synthesize surfactin under aerobic and anaerobic conditions at 25 °C, 30 °C, 37 °C and 40 °C. This genome reduced strain was named IIG-Bs20-5-1 and lacks, amongst others, genes synthesizing the lipopeptide plipastatin, the antibiotic bacilysin, toxins and prophages, as well as genes involved in sporulation. Amongst all temperatures tested, 37 °C was overall superior. In comparison to the reference strain JABs24, a surfactin synthesizing variant of *B. subtilis* 168, strain IIG-Bs20-5-1 was both aerobically and anaerobically superior with respect to specific growth rates *µ* and yields *Y*_X/S_. However, in terms of surfactin production, strain JABs24 reached higher absolute concentrations with up to 1147.03 mg/L and 296.37 mg/L under aerobic and anaerobic conditions, respectively. Concomitant, strain JABs24 reached higher *Y*_P/S_ and *Y*_P/X_. Here, an outstanding *Y*_P/X_ of 1.541 g/g was obtained under anaerobic conditions at 37 °C. The current study indicates that the employed genome reduced strain IIG-Bs20-5-1 has several advantages over the strain JABs24 such as better conversion from glucose into biomass and higher growth rates. However, regarding surfactin synthesis and yields, the strain was overall inferior at the investigated temperatures and oxygen conditions. Further studies addressing process development and strain engineering should be performed combining the current observed advantages of the genome reduced strain to increase the surfactin yields and to construct a tailor-made genome reduced strain to realize the theoretically expected advantages of such genome reduced strains.

**Electronic supplementary material:**

The online version of this article (10.1186/s13568-019-0806-5) contains supplementary material, which is available to authorized users.

## Introduction

*Bacillus subtilis* is commonly denoted the model Gram-positive bacterium. Due to its inherent characteristics, such as the natural secretion of proteases, high titers and low toxic by-product formation, this microorganism is an established bacterial platform for a variety of industrial applications (van Dijl and Hecker 2013). Amongst others, processes with *B. subtilis* as industrial host for the synthesis of proteases and riboflavin are implemented (Singh et al. 2017). Another promising metabolite is the biosurfactant surfactin, which is a cyclic lipopeptide synthesized by *Bacillus* spp. Surfactin is attributed with a variety of characteristics. These properties, ranging from exceptional surface-activity and broad spectrum physico-chemical properties, as well as antimicrobial effects, make surfactin an interesting candidate for a variety of applications such as in the agricultural, detergent and food industry (Geissler et al. 2019). However, up to date, neither a strain nor a process was described having the potential of large-scale high-titer production of surfactin as the main target product. Past research in order to increase the yields can be divided in three groups, namely (i) optimization of medium and process parameter (Freitas de Oliveira et al. 2013; Gudiña et al. 2015), (ii) applying diverse process strategies (Coutte et al. 2013; Alonso and Martin 2016) and (iii) performing strain engineering (Coutte et al. 2015; Willenbacher et al. 2016).

The complete sequence of the *B. subtilis* strain 168 genome, which comprises 4100 protein-encoding genes, was revealed by Kunst et al. (1997) and Barbe et al. (2009) resequenced this genome. Kobayashi et al. (2003) expanded the first sequence by determining essential genes in *B. subtilis* needed to sustain bacterial life. This laid the foundation of creating minimal genome cells (MGC) for *Bacillus*. MGC are defined as cells with a minimal gene set able to sustain life in the unlimited presence of nutrients and in the absence of environmental stress (Koonin 2000). Ideally, a genome reduced strain is expected to have a similar growth behavior and to yield higher product titers as the cells need less energy for genome replication, and dispensable proteins do not have to be transcribed and translated (Choe et al. 2016).

A *B. subtilis* strain derived from strain 168 with a genome reduction of 7.7% was engineered by Westers (2003). The strain lacked genes encoding for prophages as well as AT-rich islands. Under laboratory conditions, the reduction did not affect growth and viability. With respect to the heterologous production of a model protein, a positive effect of the genome reduction was not observed, and authors assumed that a redirection of energy resources into product formation did not occur. Also Ara et al. (2007) applied genome reduction to *B. subtilis* 168 in order to create a strain for the effective production of alkaline cellulase. The genome of the final strain MG1M was 0.99 Mb smaller than its parental strain and the strain showed a similar growth behavior. However, cellulase and protease activity were similar to the parental strain 168 and consequently the product titer was not increased by genome reduction. Within the same group, another genome reduced strain missing 0.87 Mb was created (Morimoto et al. 2008) and this strain was reported to have an improved extracellular and protease productivity from transformed plasmids carrying the respective genes.

Strain 168 is well-suited to employ strain engineering and was hence also used in this study as initial strain. However, strain 168 is only able to synthesize surfactin after the correction of the frameshift mutation in *sfp* (Julkowska et al. 2005; Coutte et al. 2010). To allow for better evaluation of the performed genome reduction, strain 168 with a functional *sfp*^+^ was used as reference. The employed genome reduced strain within this study also carries a functional *sfp* gene and the genome is ~ 10% smaller. Amongst others, genes encoding for plipastatin, bacylisin, toxins and prophages, as well as genes involved in sporulation, were deleted markerless by Wenzel and Altenbuchner (2015). The type-strain *B. subtilis* DSM 10^T^ was furthermore used as additional reference strain. Strain DSM 10^T^ is reported to be a natural high surfactin producing strain yielding up to 1.1 g/L surfactin in shake flask cultivation (Willenbacher et al. 2015b). In addition, for this strain, literature dealing with both the aerobic and anaerobic synthesis of surfactin was reported (Willenbacher et al. 2014, 2015a) giving a reliable comparison to results obtained within this study.

The current study aimed at evaluating the effects of genome reduction on the synthesis of surfactin in *B. subtilis* 168 with a functional *sfp* gene. To get a more fundamental background, the cultivations were performed at different temperatures and under both aerobic and anaerobic conditions. The constructed genome reduced strain was evaluated with respect to biotechnological efficiency parameters including growth rates and yields and compared to the type-strain DSM 10^T^ as well as the laboratory strain 168 carrying a functional *sfp*.

## Materials and methods

### Chemicals and materials

All chemicals used were purchased from Carl Roth GmbH & Co. KG (Karlsruhe, Germany) and were of analytical grade. The reference material for the lipopeptide surfactin (≥ 98%) was obtained from Sigma-Aldrich Laborchemikalien GmbH (Seelze, Germany).

### DNA manipulation, plasmid construction and propagation

Molecular techniques were carried out according to (Green and Sambrook 2012). The plasmids used or constructed in this study are listed in Additional file 1: Table S1 together with their construction procedure. To amplify the desired DNA fragments, polymerase chain reactions (PCRs) were performed using a polymerase (Q5^®^ High-Fidelity DNA Polymerase #M0491S, New England BioLabs^®^, Frankfurt am Main, Germany). The PCRs were run on a PCR thermal cycler (LifeECO BTC42096, Hangzhou Bioer Technology Co. Ltd., China). As a template for PCR, the chromosomal DNA (cDNA) of *B. subtilis* 168 was used unless otherwise specified. The cDNA of each strain was extracted with a DNA extraction kit (DNeasy^®^ Blood & Tissue Kit from Qiagen (Hilden, Germany) as instructed by the manufacturer. All oligonucleotides used for PCR were synthesized by Eurofins MWG Operons (Ebersberg, Germany) (Additional file 1: Table S2). DNA fragments were digested with restriction enzymes purchased from New England BioLabs^®^ (Frankfurt am Main, Germany). To purify PCR products or DNA fragments cut from agarose gel were purified employing respective kits (NucleoSpin^®^ Gel and PCR Clean-up, Macherey-Nagel GmbH, Düren, Germany). The purified DNA fragments were ligated by T4 DNA ligase (Thermo Fisher Scientific, Karlsruhe, Germany). The constructed plasmid DNAs were finally extracted (innuPREP Plasmid Mini Kit, Analytik Jena AG, Jena, Germany) and sequenced (GATC Biotech AG, Konstanz, Germany). To propagate the desired plasmids, *Escherichia coli* JM109 (Yanisch-Perron et al. 1985) was employed. The transformants of *E. coli* were selected on LB agar supplemented with 100 µg/mL ampicillin or spectinomycin according to the plasmid selection marker.

### Construction of the *B. subtilis* strains

All final strains used in this study are given in Table 1 and their construction procedure are thoroughly described in Additional file 1: Table S3. The parental strains of *B. subtilis*, namely the wild-type surfactin producing strain DSM 10^T^ and the laboratory model strain 168, were purchased from the DSMZ (German Collection of Microorganisms and Cell Cultures, Braunschweig, Germany). Transformation of *B. subtilis* strains were performed according to “Paris method” (Harwood and Cutting 1990) and the transformants were selected on LB with 100 µg/mL spectinomycin. As a parental strain for genome manipulation, the genome reduced strain IIG-Bs20-3, a derivative of strain 168 with tryptophan prototrophy and deletion of prophages and antibiotic biosynthesis genes, was used in this study (Wenzel and Altenbuchner 2015) To induce the competence in IIG-Bs20-3, a cassette containing *comK* and *comS* under control of the mannitol-inducible promoter (*mtlA*) was inserted at the 3′-end of the histidine biosynthesis operon as reported by Rahmer et al. (2015) to generate strain IIG-Bs20-5. Markerless integration of the P_*mtlA*_-*comKS* cassette was performed based on a histidine auxotrophy system developed by Motejadded and Altenbuchner (2007). To enable the production of surfactin in the desired strains, the frameshift mutation in *sfp* (shown as sfp*) was removed by transformation of the cells with pJOE8949.1. Plasmid pJOE8949.1 was an integrative plasmid carrying the functional copy of *sfp*. The gene integration was performed based on selection with spectinomycin and anti-selection on LB medium with mannose according to the method of the mannose deletion system (Wenzel and Altenbuchner 2015).

**Table 1:** Overview of strains used in the current study

Strain	Genotype or description
*B. subtilis*	
DSM10^T^	Wild-type strain^a^
JABs24	*trp*^+^ *sfp*^+^ Δ*manPA*, (Morabbi Heravi and Altenbuchner 2018) and (Reuß et al. 2017), see electronic supplementary material for detailed strain history
IIG-Bs-20-5-1	*sfp* ^+^ P_*mtlA*_-*comKS trp*^+^ Δ[SPβ] Δ[*skin*] Δ[PBSX] Δ[proΦ1] Δ[proΦ2] Δ[proΦ3] Δ[proΦ4] Δ[proΦ5] Δ[proΦ6] Δ[proΦ7] Δ[*pks*] Δ[*manPA*-*yjdF*-*yjdGHI*-*yjzHJ*] Δ[*sboAX*-*albABCDEFG*] Δ*ppsABCDE* Δ*bacABCDEF* Δ[*ytpAB*-*ytoA*] Δ[*sdpABCIR*] Δ[*bpr*-*spoIIGA*-*sigEG*] Δ[*ntdABC*-*glcP*], (Wenzel and Altenbuchner 2015), see electronic supplementary material for detailed strain history

^a^Deutsche Sammlung von Mikroorganismen und Zellkulturen GmbH (German collection of microorganisms and cell cultures)

### Cultivation conditions and preparation of culture samples

#### Mineral salt medium and flask preparation

The mineral salt medium investigated by Willenbacher et al. (2014) was used for aerobic cultivations. The final medium contained 1% glucose (*m*/*v*), 4.0 × 10^−6^ M Na_2_ EDTA × 2 H_2_O, 7.0 × 10^−6^ M CaCl_2_, 4.0 × 10^−6^ M FeSO_4_ × 7 H_2_O, 1.0 × 10^−6^ M MnSO_4_ × H_2_O, 0.1 M NH_4_Cl, 0.03 M KH_2_PO_4_, 0.04 M Na_2_HPO_4_ × 2 H_2_O and 8.0 × 10^−4^ M MgSO_4_ × 7 H_2_O. For 1 L medium, KH_2_PO_4_, Na_2_HPO_4_ × 2 H_2_O and NH_4_Cl were diluted in a total volume of 969 mL demineralized H_2_O and this solution was autoclaved after adjusting the pH to 7 with 10 M NaOH. The remaining components were added afterwards from individual stock solutions: 20 mL of an autoclaved 50% (*m*/*v*) glucose solution, 1 mL of a filter-sterilized trace element solution containing 4 × 10^−3^ M Na_2_ EDTA × 2 H_2_O, 7.0 × 10^−3^ M CaCl_2_, 4 × 10^−3^ M FeSO_4_ × 7 H_2_O, 1 × 10^−3^ M MnSO_4_ × H_2_O, and 10 mL of a MgSO_4_ solution with 8 × 10^−2^ M MgSO_4_ × 7 H_2_O. Aerobic cultivations were performed in 1 L baffled shake flasks with 100 mL medium. For anaerobic cultivations, the nitrogen source was replaced by 0.025 M NH_4_Cl and 0.1 M NaNO_3_. 96.9 mL of this solution were filled in 100 mL serum flasks. The flasks were sealed using crimp seals with a septum. Prior to autoclaving, all serum flasks were equipped with a filter in order to allow air outflow. Afterwards, 2 mL of the 50% (*m*/*v*) glucose solution, 0.1 mL of the filter-sterilized trace element stock solution and 1 mL of the MgSO_4_ stock solution were added using a sterile syringe and canula. To remove residual oxygen in the serum flasks, sterile N_2_-gas was purged through the medium for 5 min via filters.

#### Preparation of inoculum cultures

The first pre-culture was prepared by inoculating 20 mL LB medium (5 g/L tryptone, 10 g/L NaCl, 10 g/L yeast extract) with 100 µL of the respective glycerol stock in a 100 mL baffled shake flask. The shake flasks were incubated at 120 rpm and 30 °C for 24 h in an incubator shaker (Newbrunswick™/Innova^®^ 44, Eppendorf AG, Hamburg, Germany). The second pre-culture was prepared in 250 mL baffled shake flasks by diluting the respective LB-pre-culture each 1:10 and 1:20 in a final volume of 50 mL aerobic mineral salt medium. These pre-cultures were incubated for another 12 h and 24 h for the 1:10 and 1:20-dilutions, respectively.

#### Main culture

All aerobic and anaerobic cultivations were performed at 25, 30, 37 and 40 °C and 120 rpm in an incubator shaker (Newbrunswick™/Innova^®^ 44, Eppendorf AG, Hamburg, Germany). Serum flasks were incubated in a horizontal manner. For each temperature, four shake flasks and four serum flasks were prepared. Each two flasks were inoculated with a time difference of 12 h using the 1:10-dilution pre-cultures for the first set of duplicates, and the 1:20-dilution pre-cultures for the second set of duplicates. All shake flasks and serum flasks were inoculated with a resulting OD_600_ of 0.1.

### Sampling and sample analysis

Samples were taken regularly from the individual four flasks to cover every other hour of the growth phase and samples were analyzed regarding the OD_600_, glucose and surfactin concentrations. The OD_600_ was determined using a spectrophotometer (Biochrom WPA CO8000, Biochrom Ltd., Cambridge, UK). Prior to further analysis, cells were removed by centrifuging for 10 min at 4700 rpm at 4 °C (Heraeus X3R, Thermo Fisher Scientific GmbH, Braunschweig, Germany).

Surfactin was analyzed using a HPTLC system (CAMAG, Muttenz, Switzerland) with a validated method as described previously (Geissler et al. 2017). In brief, a threefold extraction of 2 mL cell-free broth with each 2 mL chloroform/methanol 2:1 (*v*/*v*) was conducted. The pooled solvent layers obtained after each extraction were evaporated to dryness in a rotary evaporator (RVC2-25 Cdplus, Martin Christ Gefriertrocknungsanlagen GmbH, Osterode am Harz, Germany) at 10 mbar and 40 °C. For HPTLC analysis, samples were resuspended in 2 mL methanol and applied as 6 mm bands on HPTLC silica gel 60 plates from Merck (Darmstadt, Germany). A surfactin standard curve was applied in the range of 30–600 ng/band. The development was conducted using chloroform/methanol/water (65:25:4, *v*/*v*/*v*) over a migration distance of 60 mm. After the development, the plate was scanned at 195 nm to quantify surfactin.

Glucose concentrations were determined using a HPTLC method as well. Proper diluted cell-free supernatants were applied as 6 mm bands and the plate was developed with acetonitrile/H_2_O (85:15, *v*/*v*) over a migration distance of 70 mm. After development, the plate was dipped in the derivatization solution diphenylamine (DPA) for 3 s and the plate was heated for 20 min at 120 °C using the TLC plate heater. DPA reagent was prepared by first dissolving 2.4 g diphenylamine and 2.4 g aniline in 200 mL methanol and then adding 20 mL 85% phosphoric acid.

For further data analysis, the OD/cell dry weight (CDW) conversion factor was determined in a pre-liminary test. Therefore, the strains were cultivated as triplicates as described above for aerobic conditions until reaching the range of maximum OD_600_. 40 mL culture were filled in dried and pre-weighted falcons and centrifuged for 10 min at 4700 rpm and 4 °C. The supernatant was discarded, and the cell pellet was washed with saline solution prior to a second round of centrifugation. After discarding the supernatant, the weight of the cell pellets were determined after drying the loaded falcons at 110 °C for 24 h and the conversion factor was calculated. In this sense, the OD/CDW conversion factor for all strains used was determined as 3.76 ± 0.17 with a %RSD of 4.47%.

### Data analysis

Biomass concentrations (g/L), glucose concentrations (g/L) and surfactin concentrations (g/L) for aerobic and anaerobic cultivations during the time course of cultivation were plotted for the individual strains and temperatures. Several process parameter were calculated for all cultivations conducted. Here, the mean values obtained for two time points were used, labelled *m*_1_ and *m*_2_. The first time point used for calculation was at 0 h of cultivation with all corresponding data. The second time point corresponded to CDW_max_. Again, all data measured at this time point were used for calculation. In addition, calculations were based on absolute values, as this compensated for the different amounts of samples taken, especially for the long lasting anaerobic cultivations. Using absolute values furthermore allowed for better comparison with literature and future planned bioreactor cultivations.

The biomass yield on substrate *Y*_X/S_ (g/g), product yield on substrate *Y*_P/S_ (g/g) and the product yield on biomass *Y*_P/X_ (g/g) were calculated using Eqs. 1, 2 and 3, respectively.

1 $$Y_{{{\text{X}}/{\text{S}}}} = \frac{{\Delta m_{\text{CDW}} }}{{\Delta m_{\text{glucose}} }}$$

2 $$Y_{{{\text{P}}/{\text{X}}}} = \frac{{\Delta m_{\text{surfactin}} }}{{\left( {\frac{{\left( {m_{{{\text{CDW}}_{1} }} + m_{{{\text{CDW}}_{2} }} } \right)}}{2}} \right)}}$$

3 $$Y_{{{\text{P}}/{\text{S}}}} = \frac{{\Delta m_{\text{surfactin}} }}{{\Delta m_{\text{glucose}} }}$$

The growth rate was calculated using Eq. 4.

4 $$\upmu = \frac{{\ln \frac{{m_{{{\text{CDW}}_{2} }} }}{{m_{{{\text{CDW}}_{1} }} }}}}{{t_{2} - t_{1} }}$$

The specific productivity *q*_spec.,surfactin_ (g_surfactin_/g_CDW_ h) was calculated using Eq. 5.

5 $$q_{{{\text{spec}}.,{\text{surfactin}}}} = \frac{{\Delta m_{\text{surfactin}} }}{{\left( {\frac{{\left( {m_{{{\text{CDW}}_{1} }} + m_{{{\text{CDW}}_{2} }} } \right)}}{2}} \right) \cdot \Delta t}}$$

In order to evaluate the reliability of the obtained data, an overall maximum relative standard deviation (RSD_max_) was determined for CDW, surfactin concentration and glucose concentration individually. Therefore, the relative standard deviations of the flasks at t_CDWmax_ were used and the mean value of the corresponding duplicates at t_0 h_. Both the mean RSD (RSD_mean_) as well as the corresponding standard deviation (SD_RSD mean_) were determined for these individual RSD. The RSD_max_ was then calculated as RSD_mean_ + SD_RSD mean_, and was determined as 12.23%, 8.53% and 0.10% for CDW, surfactin concentration and glucose concentration, respectively.

## Results

We hypothesized that the genome reduced strain *B. subtilis* IIG-Bs20-5-1 shows a similar growth behavior as compared to the reference strain *B. subtilis* JABs24 and yields higher surfactin concentrations. To get a more fundamental background on the effects of genome reduction, the cultivations were performed at four different temperatures and under both aerobic and anaerobic conditions. In addition, the high surfactin producing strain *B. subtilis* DSM 10^T^ was used as further reference strain to evaluate the overall performance of the two main strains deriving from the non-surfactin producer *B. subtilis* 168.

### Aerobic cultivations are superior to anaerobic cultivations with respect to CDW_max_ and surfactin concentration

**Fig. 1:**
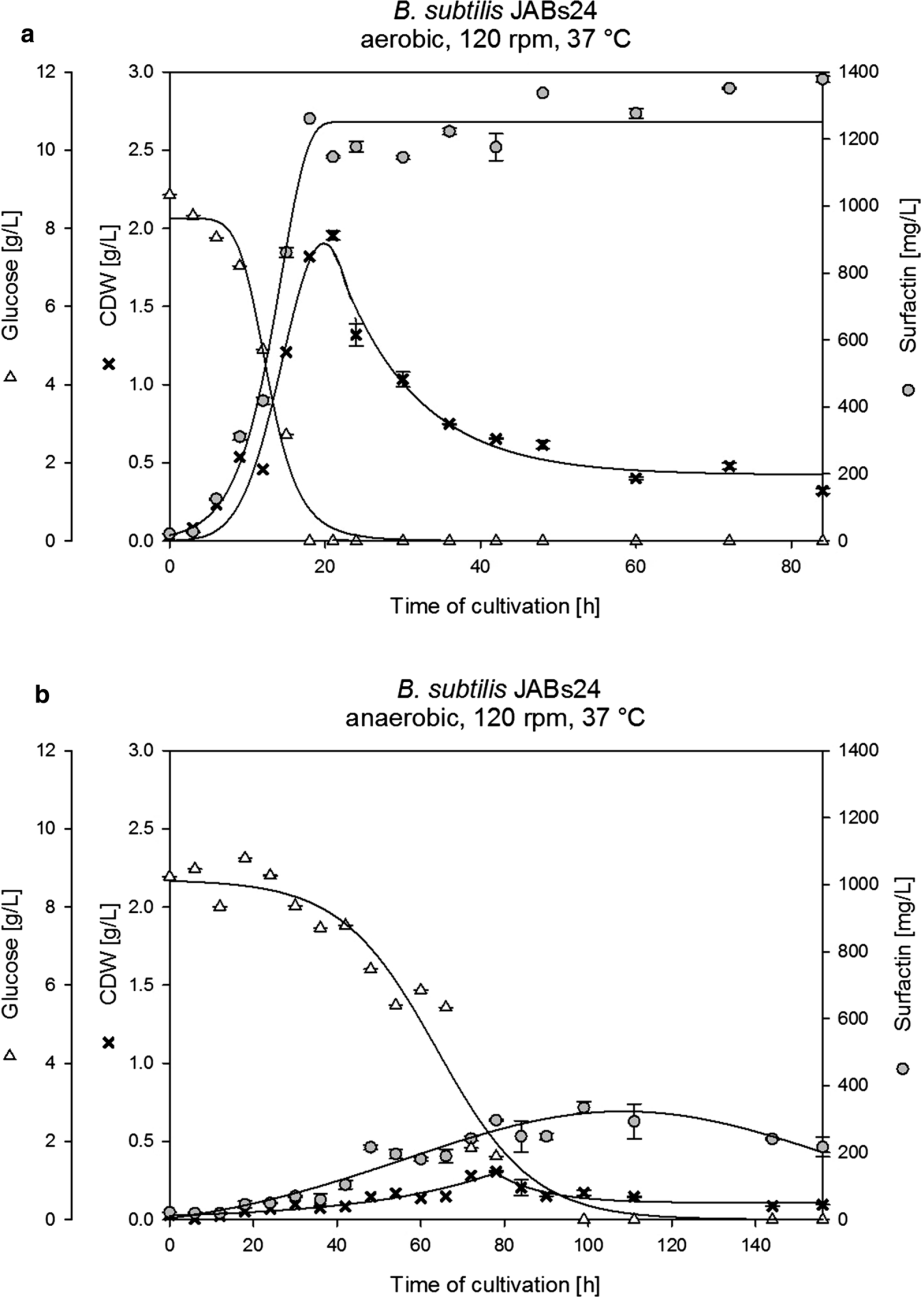
Exemplary time course of CDW (black cross) and surfactin (grey circle) in comparison to glucose (open triangle) during the cultivation of *B. subtilis* JABs24 under **a** aerobic and **b** anaerobic conditions at 37 °C

Figure 1 displays the CDW, glucose consumption and surfactin concentration during the time course of aerobic (A) and anaerobic (B) cultivation employing the strain *B. subtilis* JABs24 at 37 °C. The exemplary graphs depict the main differences for aerobic and anaerobic conditions, which are valid for all strains at all temperatures tested. The main data, including CDW_max._, surfactin_CDW max._, glucose_CDW max._, t_CDW max._, as well as the overall surfactin_max._ with the corresponding t_overall surfactin max._ are further summarized in Table 2 (aerobic) and Table 3 (anaerobic). Briefly, aerobic cultivations yielded 4.6- to 10-fold higher CDW, reached two to tenfold higher surfactin concentrations and the time of complete glucose consumption and concomitant the time to reach CDW_max_ were much shorter by factors 2 to 6 for all cultivations employed.

**Table 2:** Summary of the results obtained for aerobic cultivations at various temperatures

Aerobic shake flask cultivation							
T (°C)	*B. subtilis* strain	CDW_max_ (g/L)	Surfactin_CDW max_ (mg/L)	Glucose_CDW max_ (g/L)	t_CDW max_ (h)	Overall surfactin_max_ (mg/L)	t_overall surfactin max_ (h)
25	JABs24	1.63	766.15	0.81	54	1189.15	156
	IIG-Bs20-5-1	1.54	654.29	0.00	72	970.80	156
	DSM 10^T^	1.58	446.12	0.00	60	740.32	84
30	JABs24	1.27	602.98	0.00	36	836.23	42
	IIG-Bs20-5-1	2.12	676.46	0.00	36	740.34	144
	DSM 10^T^	1.84	209.06	0.00	30	415.79	156
37	JABs24	1.95	1147.03	0.00	21	1378.50	84
	IIG-Bs20-5-1	2.17	993.03	0.00	18	1083.92	48
	DSM 10^T^	2.10	353.93	0.00	18	793.61	60
40	JABs24	1.88	670.58	0.00	18	1079.58	24
	IIG-Bs20-5-1	2.09	681.63	0.00	18	766.72	72
	DSM 10^T^	2.04	203.45	0.00	15	406.16	84

**Table 3:** Summary of the results obtained for anaerobic cultivations at various temperatures

Anaerobic serum flask cultivation							
T (°C)	*B. subtilis* strain	CDW_max_ (g/L)	Surfactin_CDW max_ (mg/L)	Glucose_CDW max_ (g/L)	t_CDW max_ (h)	Overall surfactin_max_ (mg/L)	t_overall surfactin max_ (h)
25	JABs24	0.26	87.67	0.41	176	87.67	176
	IIG-Bs20-5-1	0.19	79.46	4.63	144	79.46	144
	DSM 10^T^	0.16	96.90	5.70	156	96.90	156
30	JABs24	0.26	209.53	5.03	90	209.53	90
	IIG-Bs20-5-1	0.41	189.64	0.00	90	189.64	90
	DSM 10^T^	0.33	76.14	6.17	90	146.64	144
37	JABs24	0.31	296.37	1.62	78	333.92	99
	IIG-Bs20-5-1	0.39	215.31	0.00	60	273.38	111
	DSM 10T	0.45	180.65	0.00	90	181.86	111
40	JABs24	0.39	75.80	2.35	42	211.24	90
	IIG-Bs20-5-1	0.26	75.96	3.25	24	158.34	90
	DSM 10^T^	0.39	110.03	1.71	90	111.56	84

Under aerobic conditions, except for strain JABs24 at 25 °C, where a glucose concentration of 0.81 g/L was measured, glucose was completely consumed under aerobic conditions when CDW_max_ was reached. For all strains, the highest CDW was reached at 37 °C with 1.95 g/L, 2.17 g/L and 2.10 g/L for strains JABs24, IIG-Bs20-5-1 and DSM 10^T^, respectively. For both IIG-Bs20-5-1 and DSM 10^T^, the lowest CDW was obtained at 25 °C with 1.54 g/L and 1.58 g/L, respectively. JABs24 yielded the lowest CDW at 30 °C with 1.27 g/L. Considering the mean CDW_max_ of the three strains at the temperatures tested, the overall %RSD of the CDW_max_ obtained was 15.94%, 12.77% and 10.71% for strains JABs24, IIG-Bs20-5-1 and DSM 10^T^, respectively. The time to reach the highest CDW was shorter the higher the temperature was. In comparison, at 25 °C the cultivation time of CDW_max_ varied between 54 and 72 h, whereas at 40 °C glucose consumption was obtained after 15 to 18 h of cultivation. Amongst the three strains tested, strain JABs24 yielded the highest surfactin concentration at 37 °C with 1147.03 mg/L. For strain IIG-Bs20-5-1, the highest concentration was also detected at 37 °C with 993.03 mg/L. Strain DSM 10^T^, however, synthesized the highest concentration of 446.12 mg/L at 25 °C, followed by 37 °C with 353.93 mg/L. During the stationary phase, surfactin concentrations further increased by a factor of 1.09 to 2.24. In general, lowest increase was monitored for strain IIG-Bs20-5-1, followed by strain JABs24 and highest increase was obtained for strain DSM 10^T^. The final overall highest concentration for the latter strain was monitored at 37 °C with an increase from 353.93 to 793 mg/L, which is lower than the highest concentrations obtained for strains JABs24 and IIG-Bs20-5-1.

As shown in Table 3, summarizing the data for anaerobic cultivations, glucose in general was not completely consumed under anaerobic conditions when CDW_max_ was reached. A trend regarding glucose consumption was not observed amongst the strains and temperatures tested. For example, for strain IIG-Bs20-5-1 with increasing temperature, 4.63 g/L, 0.00 g/L, 0.00 g/L and 3.25 g/L were measured prior to an observed decline in CDW. Similar to aerobic cultivations, the time to reach CDW_max_ was shorter the higher the temperature was set. Excluding strain DSM 10^T^, the time varied between 144 and 176 h at 25 °C, and 24 to 42 h at 40 °C. Strain DSM 10^T^, however, reached the CDW_max_ after 90 h of cultivation at 30 °C, 37 °C and 40 °C. With respect to CDW_max_, all strains reached the lowest value at 25 °C. Amongst the other temperatures tested, no trend was observed, and data varied between 0.26 and 0.45 g/L. With respect to surfactin, anaerobic cultivations showed a similar pattern as compared to aerobic cultivations. At 37 °C, all strains synthesized the highest amounts of surfactin with 296.37 g/L, 215.31 g/L and 180.65 g/L at CDW_max_. At 25 °C, the overall surfactin_max_ matched the concentration at CDW_max_. A similar result was obtained for strains JABs24 and IIG-Bs20-5-1 at 30 °C. The highest increase of surfactin was detected for strain DSM 10^T^ at 30 °C, as well as for strains JABs24 and IIG-Bs20-5-1 at 40 °C where the concentration reached values of 1.93- to 2.79-fold higher. Considering the overall highest surfactin concentration, strain DSM 10^T^ was, similar to aerobic cultivations, inferior to strain JABs24 yielding the highest values.

### Strain IIG-Bs20-5-1 yields higher growth rates *µ* and *Y*_X/S_ than strain JABs24

**Fig. 2:**
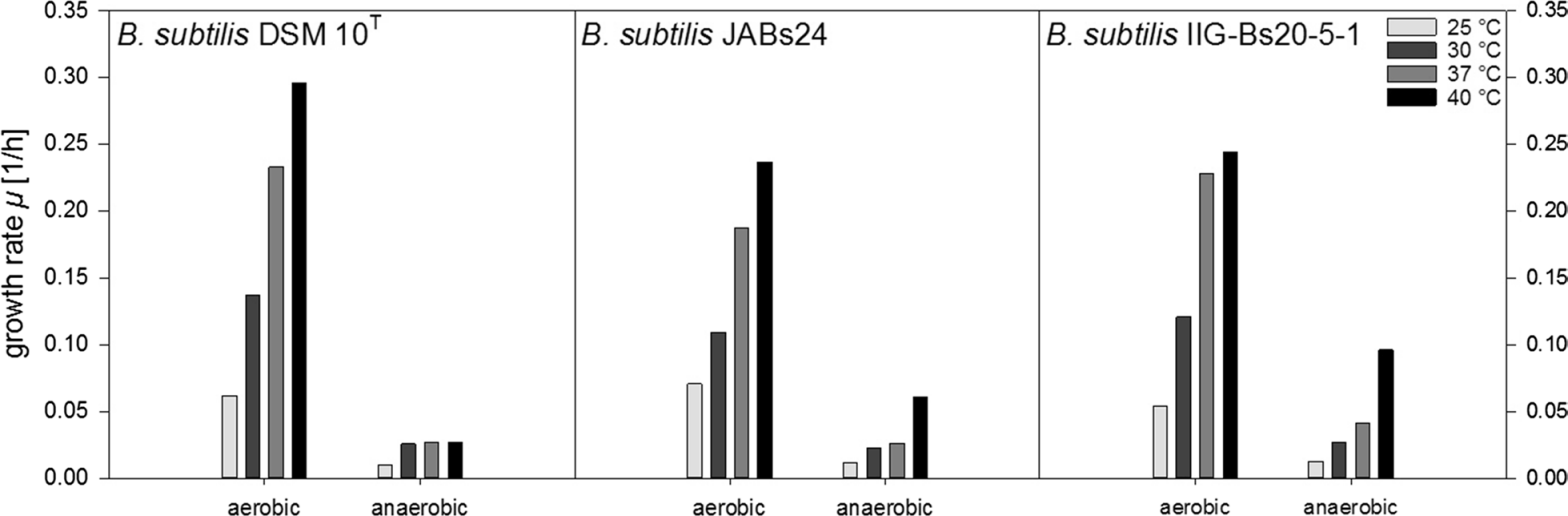
Overview of the calculated growth rates *µ* (1/h) for strains *B. subtilis* DSM 10^T^, *B. subtilis* JABs24 and *B. subtilis* IIG-Bs20-5-1 under aerobic and anaerobic conditions at 25 °C, 30 °C, 37 °C and 40 °C

As illustrated in Fig. 2, the growth rates *µ* (1/h) increased with increasing temperature for all strains tested under both aerobic and anaerobic conditions. In accordance to the higher CDW_max_ reached and the reduced time to reach CDW_max_, aerobic cultivations possessed higher growth rates. For example, at 25 °C and 40 °C, strain JABs24 reached a growth rate of 0.070 1/h and 0.236 1/h under aerobic, and of 0.012 1/h and 0.061 1/h under anaerobic conditions. Strain DSM 10^T^ yielded higher growth rates under aerobic conditions than the other two strains except at 25 °C. The genome reduced strain furthermore performed better than strain JABs24, albeit the exception of 25 °C. At this condition, JABs24 was superior to both other strains. Under anaerobic conditions, strain IIG-Bs20-5-1 surpassed the growth rates for JABs24 and DSM 10^T^ at all temperatures tested, with the highest growth rate obtained at 40 °C with 0.096 1/h and the lowest at 25 °C with 0.013 1/h.

**Fig. 3:**
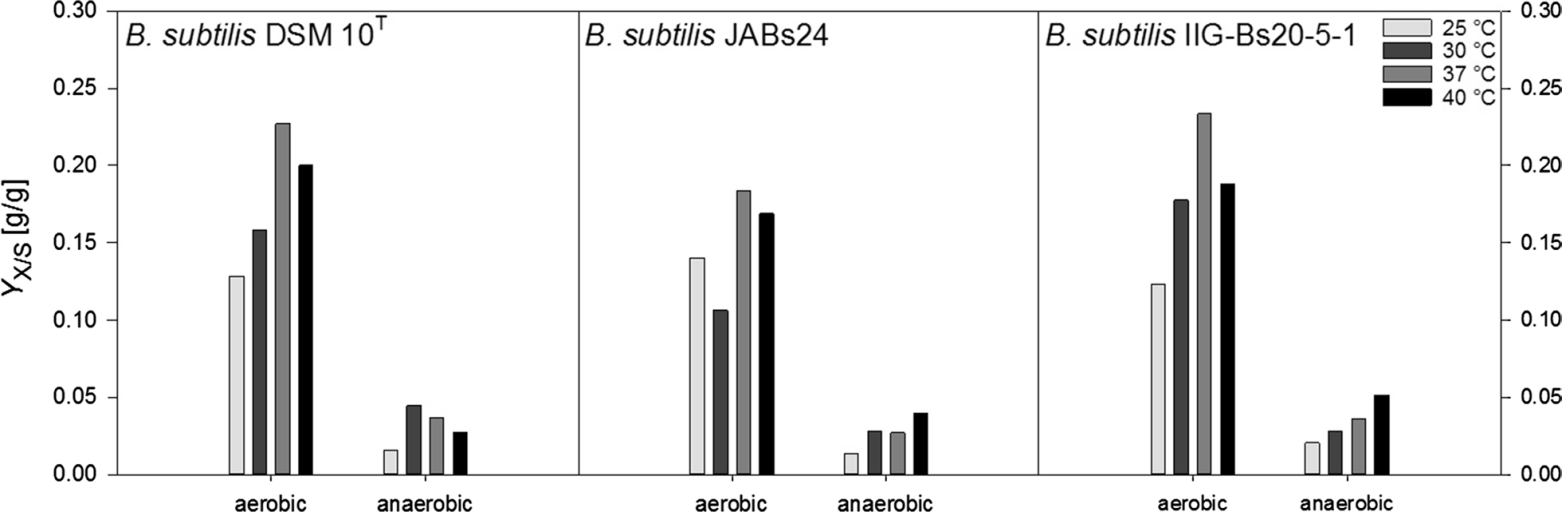
Overview of the calculated biomass yields on substrate *Y*_X/S_ (g/g) for strains *B. subtilis* DSM 10^T^, *B. subtilis* JABs24 and *B. subtilis* IIG-Bs20-5-1 under aerobic and anaerobic conditions at 25 °C, 30 °C, 37 °C and 40 °C

Figure 3 depicts the results obtained for the biomass yield on substrate *Y*_X/S_ (g/g). Under aerobic conditions, the highest conversion from glucose into biomass was obtained at 37 °C with yields of 0.184 g/g, 0.234 g/g and 0.227 g/g for strains JABs24, IIG-Bs20-5-1 and DSM 10^T^, respectively. Except for strain JABs24 at 25 °C, values increased with increasing temperature and after reaching a maximum at 37 °C, yields declined at 40 °C. Though, values at 40 °C were still higher than at 30 °C. Under anaerobic conditions, *Y*_X/S_-values were much lower and did not surpass 0.051 g/g, which was obtained for strain IIG-Bs20-5-1 at 40 °C. In comparison to the data determined for the aerobic cultivations, a similar trend or strain possessing generally the highest conversion at the temperatures tested cannot be emphasized.

### Strain JABs24 yields outstanding values for *Y*_P/X_ with the genome reduced strain being slightly inferior

**Fig. 4:**
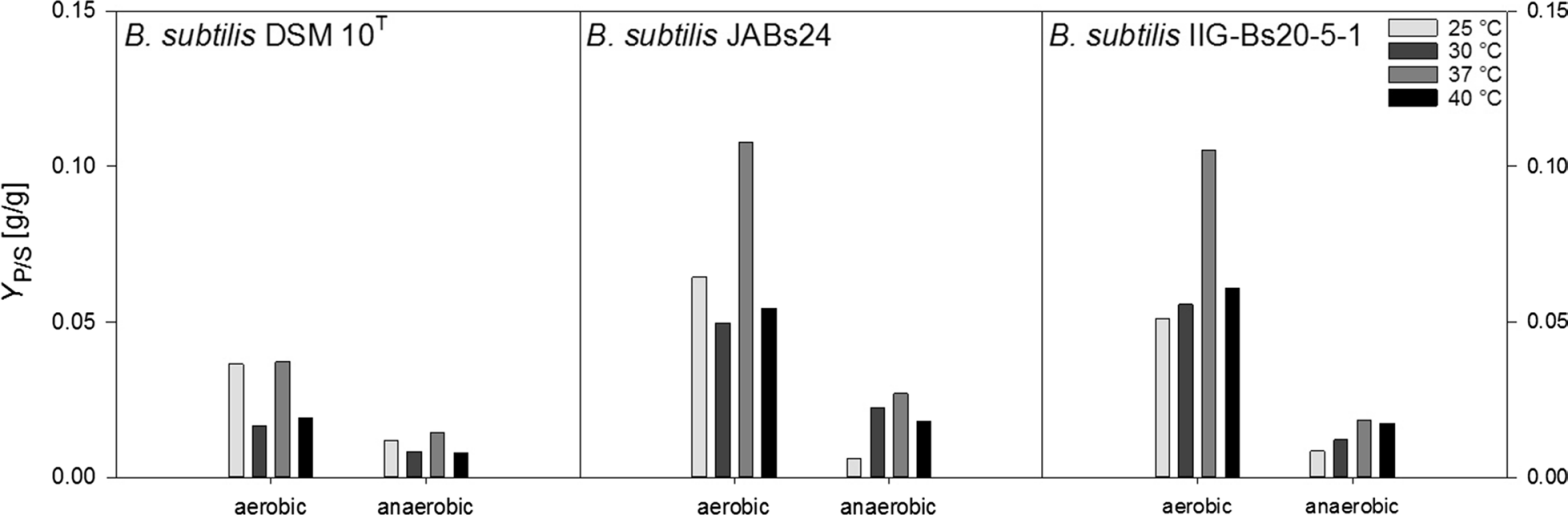
Overview of the calculated product yields on substrate *Y*_P/S_ (g/g) for strains *B. subtilis* DSM 10^T^, *B. subtilis* JABs24 and *B. subtilis* IIG-Bs20-5-1 under aerobic and anaerobic conditions at 25 °C, 30 °C, 37 °C and 40 °C

Figure 4 displays the product yield per substrate *Y*_P/S_ (g/g) for the different temperatures tested. 37 °C was superior under both aerobic and anaerobic conditions. Similar to the growth rate *µ* and the *Y*_X/S_, aerobic calculated *Y*_P/S_ were much higher than anaerobic values. The highest *Y*_P/S_ were obtained under aerobic conditions at 37 °C for strain JABs24 with 0.108 g/g and for strain IIG-Bs20-5-1 with 0.105 g/g. These values were about twofold higher than at the other temperatures tested. Under anaerobic cultivations, the *Y*_P/S_ at 37 °C reached 0.027 g/g and 0.018 g/g, respectively for these two strains. The variation of the *Y*_P/S_ was overall lower than under aerobic conditions. The *Y*_P/S_ for strain DSM 10^T^ did not surpass 0.037 g/g under aerobic, and 0.015 g/g under anaerobic conditions, both obtained at 37 °C, which correlates to the overall lower obtained surfactin concentrations.

**Fig. 5:**
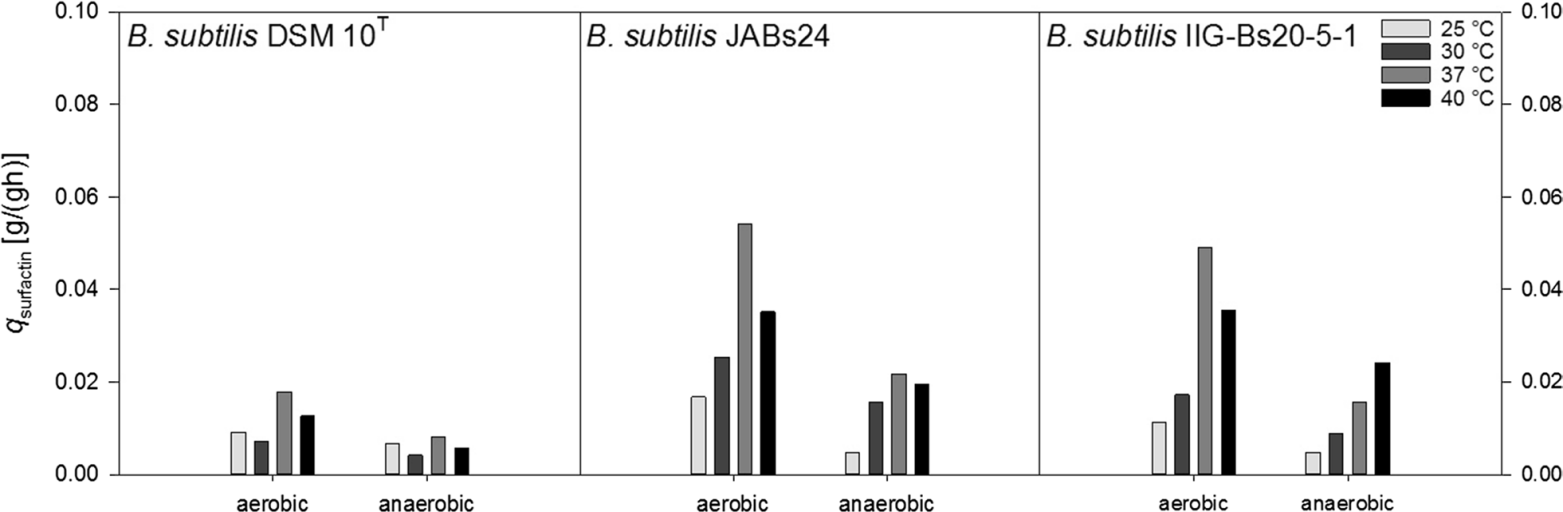
Overview of the calculated surfactin productivities *q*_surfactin_ (g/g h) for strains *B. subtilis* DSM 10^T^, *B. subtilis* JABs24 and *B. subtilis* IIG-Bs20-5-1 under aerobic and anaerobic conditions at 25 °C, 30 °C, 37 °C and 40 °C

Figure 5 illustrates the specific productivity *q*_surfactin_ (g/g h). With respect to the long cultivation times under anaerobic conditions, the values calculated were rather low. Here, a trend could be observed for strain IIG-Bs20-5-1, and the overall productivity increased from 0.005 to 0.024 g/g h with increasing temperature. For strain JABs24, highest productivity was obtained at 37 °C with 0.022 g/g h. Strain DSM 10^T^ showed the overall lowest productivity and values did not exceed 0.008 g/g h. Under aerobic conditions, 37 °C was superior for all strains tested and productivities of 0.054 g/g h, 0.049 g/g h and 0.018 g/g h were recorded for strains JABs24, IIG-Bs20-5-1 and DSM 10^T^, respectively. 40 °C was furthermore superior to 30 °C, similar to the observations for the *Y*_X/S_.

**Fig. 6:**
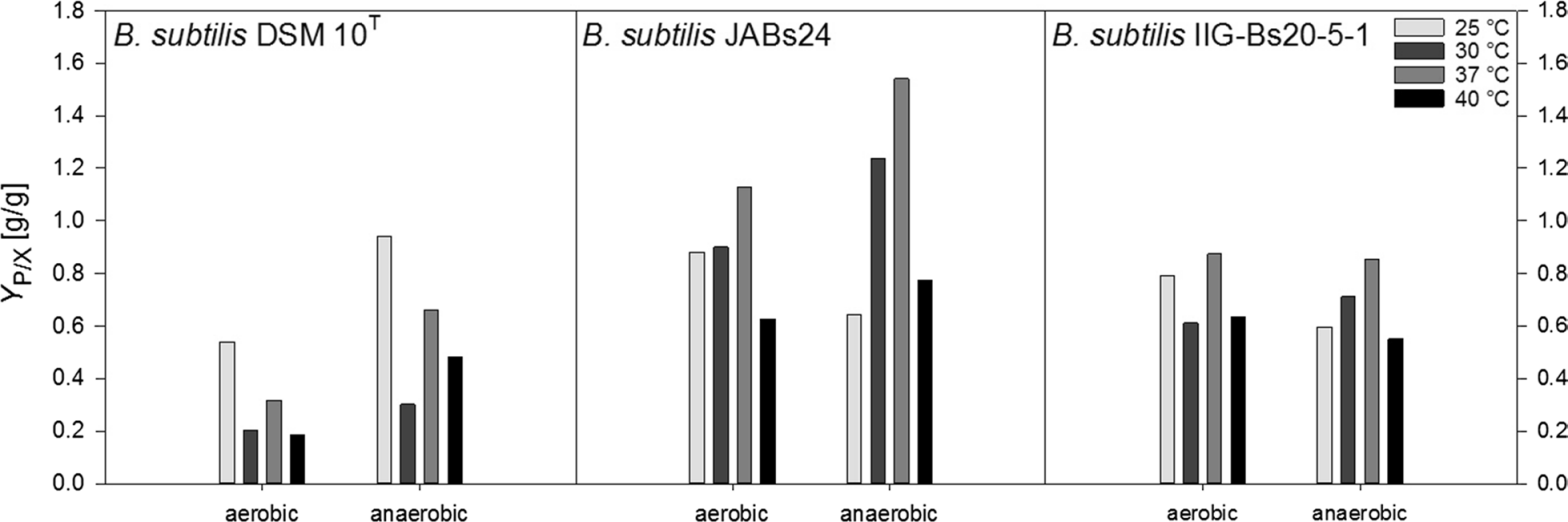
Overview of the calculated product yields on biomass *Y*_P/X_ (g/g) for strains *B. subtilis* DSM 10^T^, *B. subtilis* JABs24 and *B. subtilis* IIG-Bs20-5-1 under aerobic and anaerobic conditions at 25 °C, 30 °C, 37 °C and 40 °C

Figure 6 shows the product yields per biomass *Y*_P/X_ (g/g). The *Y*_P/X_ for strain DSM 10^T^ obtained at 25 °C surpassed the values at the other three temperatures tested and reached 0.541 g/g and 0.942 g/g under aerobic and anaerobic conditions, respectively. 30 °C yielded the lowest values, and 37 °C was furthermore superior to 40 °C. For strain DSM 10^T^ the *Y*_P/X_ was 1.48- to 2.58-fold higher under anaerobic conditions at all temperatures run. For strain JABs24, yields obtained anaerobically were 1.02- to 1.32-fold higher at 30 °C to 40 °C, but 0.73-fold lower at 25 °C. The highest yield was determined anaerobically at 37 °C with 1.541 g/g. The overall lowest yield was determined aerobically at 25 °C with 0.627 g/g. Yields determined for the genome reduced strain IIG-Bs20-5-1 were overall slightly lower than for strain JABs24, but in general surpassed the *Y*_P/X_ of strain DSM 10^T^. However, other than for the two reference strains, differences in aerobic and anaerobic yields were not as significant and the *Y*_P/X_ was mostly inferior under anaerobic conditions. For example, the *Y*_P/X_ were 0.75- to 0.97-fold lower at 25 °C, 37 °C and at 40 °C. At 30 °C, the *Y*_P/X_ under aerobic and anaerobic conditions were 0.611 g/g and 0.712 g/g, respectively. The overall highest *Y*_P/X_ were obtained at 37 °C with 0.873 g/g and 0.854 g/g for aerobic and anaerobic cultivations.

## Discussion

The current research aimed at investigating the suitability of a genome reduced strain to produce surfactin. We hypothesized that the surfactin yields in strain *B. subtilis* IIG-Bs20-5-1 benefit from the employed genome reduction, and that the strain shows a similar growth pattern than the primary reference strain JABs24.

### Evaluation of growth, surfactin synthesis and yields obtained under the conditions tested

The growth behavior of strains 168 *sfp*^0^ and DSM 10^T^ was reported to be similar by Kabisch et al. (2013) and Julkowska et al. (2005). For specific growth rates, both strains yielded ~ 0.450 1/h (Kabisch et al. 2013), and strain BBG258, a 168 *sfp*^+^ derivative, yielded 0.499 1/h in a study conducted by Dhali et al. (2017). However, although the aerobic growth rates obtained within this study were much lower with < 0.234 1/h, it must be noted that strain 168 varies between laboratories due to domestication, and DSM 10^T^ is reported to have different variants as well, such as ATCC 6051 (Kabisch et al. 2013) and NCBI 3610 (Julkowska et al. 2005). Hence, the assignment remains a challenge and comparisons are hindered (Zeigler et al. 2008). Contrariwise to literature, considering the overall growth rates obtained within this study at 30 °C, 37 °C and 40 °C, strain DSM 10^T^ was superior to strain JABs24 under aerobic conditions. It might be that the restoration of *sfp* led to a reduced growth rate, as secondary metabolites such as lipopeptides are thought to be metabolically demanding as they are synthesized by large non-ribosomal peptide synthetases (Fischbach and Walsh 2006). In addition, *B. subtilis* undergoes a complex machinery of cell differentiation with surfactin being an important signal molecule (López and Kolter 2010). Reestablishing of surfactin synthesis is expected to change the overall cellular differentiation process, as reported by Julkowska et al. (2005) where surfactin induced swarming of cells. Interestingly, under anaerobic conditions strain DSM 10^T^ was inferior to JABs24 and the genome of JABs24 hence might be able to better adapt to anaerobic conditions.

Next to the growth rate, data obtained for the surfactin concentrations for strains JABs24 (Ongena et al. 2007; Coutte et al. 2010) and DSM 10^T^ (Willenbacher et al. 2015b), as well as for the anaerobic yields calculated for strain DSM 10^T^ are well in accordance to literature (Willenbacher et al. 2015a). However, next to the genetic alterations of strains used in different studies, comparisons are also difficult when different media or process set-ups and parameter are used as in Willenbacher et al. (2014). In this study, strain DSM 10^T^ was cultivated in a 2.5 L benchtop bioreactor under aerobic conditions with surfactin recovery employing foam fractionation. *Y*_P/X_, *Y*_X/S_ and *Y*_P/S_ obtained were 0.192 g/g, 0.268 g/g and 0.052 g/g. In the current study, at 30 °C, the *Y*_P/X_ was in a similar range with 0.204 g/g, and both *Y*_X/S_ and *Y*_P/S_ were much lower with 0.158 g/g and 0.017 g/g, respectively.

Also with respect to the temperature, different strains, media and parameter used make it difficult to draw meaningful conclusions. Nevertheless, within this study, 37 °C was overall superior for all parameter tested and amongst the strains cultivated, which is well in accordance to different literature (Sen and Swaminathan 1997; Rahman and Ano 2009; Ghribi et al. 2012; Meena et al. 2018).

Davis et al. (1999) examined different batch cultures and reported that the highest *Y*_P/X_ was obtained under nitrate-limited oxygen-depleted conditions with 0.075 g/g. In comparison, aerobic nitrogen-limited conditions yielded only 0.021 g/g. However, although the current results also indicated that, regardless nitrogen-limitation, anaerobic cultivations yielded higher *Y*_P/X_, literature often reports that surfactin production is favored at good oxygenation (Yeh et al. 2006; Abdel-Mawgoud et al. 2008; Fahim et al. 2012; Ha et al. 2018). Although the *Y*_P/X_ is often not calculated, data presented by Abdel-Mawgoud et al. (2008) also indicated that the *Y*_P/X_ is increased at higher aeration in a medium containing only nitrate as sole nitrogen source. In the current study the *Y*_P/X_ was generally superior under anaerobic conditions, but it has to be further elucidated which mechanisms are causing this effect. For example, the presence of nitrate itself and the concomitant nitrate respiration might result in the overall higher *Y*_P/X_. As *B. subtilis* is also able to grow aerobically on nitrate as sole nitrogen source, further studies are desirable to evaluate the effect of the nitrogen source nitrate, especially as *B. subtilis* contains two nitrate reductases, with NasBC being active both aerobically and anaerobically in the assimilatory pathway, and NarGHJ being only induced anaerobically in the presence of the alternative electron acceptor nitrate (Nakano et al. 1998; Ye et al. 2000).

To sum up, the results obtained within the current study are comparably reliable and especially the high *Y*_P/X_ of 1.127 g/g and 1.541 g/g obtained for strain JABs24 under both aerobic and anaerobic conditions at 37 °C must be emphasized which surpasses many reported *Y*_P/X_ in literature for different process set-ups (Davis et al. 1999; Chtioui et al. 2012; Willenbacher et al. 2014, 2015a; Coutte et al. 2015; Dhali et al. 2017). Coutte et al. (2010) also reached a high *Y*_P/X_ of 1.08 g/g in a 168 *sfp*^+^ derivative strain, but it has to be pointed out that a functional *sfp* from *B. subtilis* ATCC 21332 was integrated instead of restoration of the original *sfp* as performed in this study. In this sense, with the *Y*_P/X_ being superior for strain JABs24, the most crucial question is now, if the genome reduction led to a benefit as hypothesized.

### Strains JABs24 vs. IIG-Bs20-5-1—evaluation of the effect of genome reduction

Constructing genome reduced strains is an interesting technique. The designed strains ideally show a similar or even better growth behavior than the parental strain (Choe et al. 2016). In addition, when a strain shall be used as catalyst for the synthesis of a specific product, genome reduced strains are expected to yield higher titers as the deletion of non-essential genes saves metabolic resources (Choe et al. 2016). In 2008, the suitability of genome reduced *B. subtilis* with the aim of producing a target product was reported by Morimoto et al. (2008). However, the product used within that study was synthesized from a plasmid carrying the respective gene. In the current study, the target product surfactin is naturally produced by *B. subtilis* and the synthesis is based on a complex quorum sensing system (Hamoen 2003). Dhali et al. (2017) reported an increase in surfactin yield from 527 ± 80 to 1556 ± 123 mg/g CDW by deleting the gene encoding for CodY in a 168 *sfp*^+^ derivative. In our study, we expected an increase in surfactin synthesis as, for example, (i) the gene *pps*, which encodes for the large non-ribosomal peptide synthetase of the lipopeptide plipastatin was deleted and the cell can hence save resources, (ii) sporulation factors were deleted which influence cell differentiation and a higher ratio of cells might become surfactin producers, (iii) prophages were deleted which is expected to, amongst others, reduce the formation of autolysins and hence cell lysis.

A strain with several identical deletions was constructed by Westers (2003) and they reported that the strain possessing a 7.7% smaller genome exhibited a comparable growth behavior and biomass yield on substrate under laboratory conditions as the reference strain. However, they also pointed out that the suitability of their constructed genome reduced strain as bacterial cell factory remains open. In our study, the genome reduced strain IIG-Bs20-5-1 yielded either similar or even higher CDW_max_, but especially under anaerobic conditions, the time of cultivation to reach CDW_max_ was shorter than for strain JABs24. This observation is further on reflected in the determined growth rate *µ*, which is overall both aerobically and anaerobically higher for strain IIG-Bs20-5-1. The *Y*_X/S_ of strain IIG-Bs20-5-1 also surpassed the respective yields of strain JABs24 at almost all conditions tested, indicating a better conversion efficiency from glucose into biomass. Comparing the data for the growth rate *µ*, *Y*_X/S_ and the time to reach CDW_max_ of strain JABs24 and IIG-Bs20-5-1, it gives the idea that the employed genome reduction has a higher positive impact especially under anaerobic conditions and at 37 °C. However, the better growth and the obtained higher biomass did not lead to higher surfactin concentrations and product yields per biomass *Y*_P/X_. In both parameter, strain JABs24 was superior. With some exceptions, aerobically at 30 °C and 40 °C, and anaerobically at 40 °C, strain IIG-Bs20-5-1 yielded higher surfactin concentrations at CDW_max_. However, apart from the surfactin concentration at CDW_max_, the overall surfactin_max_ was higher for JABs24 throughout all experiments. The *Y*_P/X_ for strain JABs24 was beyond that generally higher under anaerobic conditions, which is well in accordance to the observations from Willenbacher et al. (2015a). Interestingly, the *Y*_P/X_ was in a similar range for strain IIG-Bs20-5-1 under aerobic and anaerobic conditions at the respective temperatures, indicating that the genome reduction has a rather negative effect on the surfactin synthesis under anaerobic conditions. In this context, the employed genome reduction led to improved growth rates and *Y*_X/S_, but did neither positively influence the surfactin synthesis nor productivity, nor the yields *Y*_P/S_ and *Y*_P/X_.

To sum up, aerobic cultivations were superior with respect to *µ*, *Y*_X/S_ and *Y*_P/S_, while anaerobic cultivations overall yielded better *Y*_P/X_, especially for strain JABs24. In comparison to the reported high surfactin producing wild-type DSM 10^T^, both strain JABs24 as well as the genome reduced strain IIG-Bs20-5-1 reached excellent values for surfactin concentration and *Y*_P/X_. In direct comparison, the genome reduced strain was superior to its main reference strain with respect to both the growth rate *µ* and *Y*_X/S_. Anaerobic conditions furthermore favored the growth and conversion from glucose into biomass for IIG-Bs20-5-1 compared to strain JABs24, however, the surfactin synthesis was negatively affected anaerobically. Although hypothesized that the genome reduced strain leads to better surfactin yields, strain JABs24 reached outstanding surfactin concentrations aerobically and *Y*_P/X_ values anaerobically.

## Additional file

**Additional file 1: Table S1.** Plasmids used in this study. **Table S2.** Oligonucleotides used in this study. **Table S3.** Strains used in this study.

## Data Availability

Data relevant to this study which is not included in the manuscript is available as additional file. Please turn to the corresponding author for all other requests.
