# Supplementary material for: Evaluation of surfactin synthesis in a genome reduced Bacillus subtilis strain
Source: AMB Express. 2019 Jun 12;9:84. doi: 10.1186/s13568-019-0806-5 (PMC6562014; doi:10.1186/s13568-019-0806-5)
Supplement: Supplementary file 1 — Additional file 1: Table S1. Plasmids used in this study. Table S2. Oligonucleotides used in this study. Table S3. Strains used in this study. [file 13568_2019_806_MOESM1_ESM.docx]

**Additional files**

**Evaluation of Surfactin Synthesis in a Genome Reduced *Bacillus subtilis* Strain**

Mareen Geissler ^a^, Ines Kühle ^a^, Kambiz Morabbi Heravi ^a^, Marius Henkel ^a, *^, Josef Altenbuchner ^b^, Rudolf Hausmann ^a^

^a^ Institute of Food Science and Biotechnology (150), Department of Bioprocess Engineering (150k), University of Hohenheim, Fruwirthstr. 12, 70599 Stuttgart, Germany

^b^ Institut für Industrielle Genetik, Universität Stuttgart, Allmandring 31, 70569 Stuttgart, Germany

*****Corresponding author:

Address: Fruwirthstr. 12, 70599 Stuttgart, Germany

Phone: +49-711-459-24726

Fax: +49-711-459-24722

Email: marius.henkel@uni-hohenheim.de

**TABLE S1** Plasmids used in this study

| Plasmid | Genotype or description | Vector  (cut) | Insert  (cut) | Reference, precursor, or method of construction |
| --- | --- | --- | --- | --- |
| pHM30 | *ori*_pUC18_, *bla*, ′*hisF*-*hisI*′-*spcR*-′*yvcA*-*yvcB*′ |  |  | Motejadded and Altenbuchner (2007) |
| pJOE7361.1 | *ori*_pUC18_, *bla*, *yvcB*′-P*_mtlA_*-*comK*-*comS*-*hisI*-*hisF*′ |  |  | Rahmer et al. (2015) |
| pJOE8739.1 | *ori*_pBR322_, *rop*, *spcR*, P*_manP_*-*manP*, *rha*P*_BAD_*-*ccdB* |  |  | Reuß et al. (2017) |
| pJOE8940.1 | *ori*_pBR322_, *rop*, *spcR*, P*_manP_*-*manP*, *sfp** | pJOE8739.1  (SmaI) | PCR  s10550-s10551 | *sfp** → pJOE8739.1 |
| pJOE8949.1 | *ori*_pBR322_, *rop*, *spcR*, P*_manP_*-*manP*, *sfp* | pJOE8940.1  Site directed mutagenesis  s10718-s10719 |  | *sfp** → *sfp* |

**TABLE S2** Oligonucleotides used in this study

| Name | Sequence (5' → 3') | Application |
| --- | --- | --- |
| **s10550** | aaa aaa ccc ggg atg aag att tac gga att tat atg | Amplification of *sfp** |
| **s10551** | aaa aac ccg ggg cgc tga ctg aca cgt tct | Amplification of *sfp** |
| **s10718** | caa aca gga agg caa agg ctt atc gct t | Site-directed mutagenesis of *sfp* |
| **s10719** | ata aaa ctt tct ttc att gac cat aga tga ta | Site-directed mutagenesis of *sfp* |

**TABLE S3** Strains used in this study

| Strain | Genotype or description | Reference, precursor, or method of construction |
| --- | --- | --- |
| ***E. coli* K12** |  |  |
| JM109 | *recA1*,*endA1*,*gyrA96*,*thi-1*,*hsdR17*(r_K_^-^, m_k_^+^),*mcrA*, *supE44*,*gyrA96*, *relA1,* λ^–^, Δ(*lac*-*proAB*)*,* F' (*traD36*,*proAB*^+^,*lacI^q^*, (Δ*lacZ*)M15) | (Yanisch-Perron et al. 1985) |
| ***B. subtilis*** |  |  |
| DSM10^T^ | Wild-type strain | DSMZ^α^ (#DSM10^T^) |
| 168 | *trpC*2 | DSMZ (#DSM23778) |
| KM297 | Δ*manPA* | (Morabbi Heravi and Altenbuchner 2018) |
| JABs24 | *trp*^+^ *sfp*^+^ Δ*manPA* | pJOE8949.1 → KM297 |
| IIG-Bs-20-3 | *trp*^+^  Δ[SPβ] Δ[*skin*] Δ[PBSX] Δ[proΦ1] Δ[proΦ2] Δ[proΦ3] Δ[proΦ4] Δ[proΦ5] Δ[proΦ6] Δ[proΦ7] Δ[*pks*] Δ[*manPA*-*yjdFGHI-yjzH-yjdJ*] Δ[*sboAX*-*albABCDEFG*] Δ*ppsABCDE* Δ*bacABCDEF* Δ[*ytpAB*-*ytoA*] Δ[*sdpABCIR*] Δ[*bpr*-*spoIIGA*-*sigEG*] Δ[*ntdABC*-*glcP*] | (Wenzel and Altenbuchner 2015) |
| IIG-Bs20-3 pHM30 | Δ*hisI*::*spcR*  *trp*^+^ Δ[SPβ] Δ[*skin*] Δ[PBSX] Δ[proΦ1] Δ[proΦ2] Δ[proΦ3] Δ[proΦ4] Δ[proΦ5] Δ[proΦ6] Δ[proΦ7] Δ[*pks*] Δ[*manPA*-*yjdFGHI-yjzH-yjdJ*] Δ[*sboAX*-*albABCDEFG*] Δ*ppsABCDE* Δ*bacABCDEF* Δ[*ytpAB*-*ytoA*] Δ[*sdpABCIR*] Δ[*bpr*-*spoIIGA*-*sigEG*] Δ[*ntdABC*-*glcP*] | pHM30 → IIG-Bs-20-3 |
| IIG-Bs-20-5 | P*_mtlA_*-*comKS*  *trp*^+^ Δ[SPβ] Δ[*skin*] Δ[PBSX] Δ[proΦ1] Δ[proΦ2] Δ[proΦ3] Δ[proΦ4] Δ[proΦ5] Δ[proΦ6] Δ[proΦ7] Δ[*pks*] Δ[*manPA*-*yjdF-yjdGHI-yjzHJ*] Δ[*sboAX*-*albABCDEFG*] Δ*ppsABCDE* Δ*bacABCDEF* Δ[*ytpAB*-*ytoA*] Δ[*sdpABCIR*] Δ[*bpr*-*spoIIGA*-*sigEG*] Δ[*ntdABC*-*glcP*] | pJOE7361.1 → IIG-Bs-20-3 pHM30 |
| IIG-Bs-20-5-1 | *sfp*^+^  P*_mtlA_*-*comKS* *trp*^+^ Δ[SPβ] Δ[*skin*] Δ[PBSX] Δ[proΦ1] Δ[proΦ2] Δ[proΦ3] Δ[proΦ4] Δ[proΦ5] Δ[proΦ6] Δ[proΦ7] Δ[*pks*] Δ[*manPA*-*yjdF-yjdGHI-yjzHJ*] Δ[*sboAX*-*albABCDEFG*] Δ*ppsABCDE* Δ*bacABCDEF* Δ[*ytpAB*-*ytoA*] Δ[*sdpABCIR*] Δ[*bpr*-*spoIIGA*-*sigEG*] Δ[*ntdABC*-*glcP*] | pJOE8949.1 → IIG-Bs-20-5 |

^α^ Deutsche Sammlung von Mikroorganismen und Zellkulturen GmbH (German collection of microorganisms and cell cultures).
